# Dietary intake and plasma levels of polyunsaturated fatty acids in early-stage Parkinson’s disease

**DOI:** 10.1038/s41598-021-92029-x

**Published:** 2021-06-14

**Authors:** Dallah Yoo, Yunsook Lim, Yiseul Son, Hyunkyung Rho, Chaewon Shin, Tae-Beom Ahn

**Affiliations:** 1 Department of Neurology, Kyung Hee University Hospital, Kyung Hee University College of Medicine, Seoul, Republic of Korea; 2 Department of Food and Nutrition, College of Human Ecology, Kyung Hee University, Seoul, Republic of Korea; 3 Department of Neurology, College of Medicine, Chungnam National University, Daejeon, Republic of Korea; 4 Department of Neurology, Neuroscience Center, Chungnam National University Sejong Hospital, Sejong, Republic of Korea

**Keywords:** Diseases, Medical research, Neurology, Signs and symptoms

## Abstract

Polyunsaturated fatty acids (PUFA) are important for neuronal function and may contribute to the development of neurodegenerative diseases. Here, we investigated the correlation between dietary intake and plasma concentrations of PUFA and their associations with clinical severity in early-stage Parkinson’s disease (PD). In a case–control study with 38 patients with PD and 33 controls, we assessed dietary intake using food frequency questionnaires and simultaneously measured the plasma levels of five PUFA. No differences were observed in dietary total energy and lipid intake, including PUFA, between patients with PD and controls. However, α-linolenic acid (ALA), linoleic acid (LA), and arachidonic acid (AA) plasma levels were lower in patients with PD. The association between dietary intake and plasma PUFA concentrations was not significant in patients with PD. ALA and LA plasma levels were inversely correlated with motor severity in patients with PD, while docosahexaenoic acid and AA plasma levels were positively correlated with non-motor symptoms after controlling for age and sex.

## Introduction

Parkinson’s disease (PD) is the second most common neurodegenerative disease, affecting approximately 1% of people aged 65 years or older. Environmental factors are important in the pathogenesis of PD, specifically in patients older than 50 years^1^. Daily food intake is studied as an important environmental factor^2^.

The role of polyunsaturated fatty acids (PUFA) in PD has been widely studied in terms of molecular pathophysiology^3^, epidemiological risk factors^4^, and neuroprotective effects^5^. Abundant PUFA in neural plasma membranes could be sources of oxygen radicals through lipid peroxidation^6^ and may promote α-synuclein (αSyn) oligomerization with further aggregation in vitro and in vivo mouse models^3, 7^. Clinical studies assessing the association between PUFA intake and PD risk remain controversial^4, 8–10^. Direct analyses of lipid metabolites have shown decreased PUFA levels in PD^11, 12^. In animal studies, n-3 PUFA has shown neuroprotective effects on dopaminergic neurons and possibly promotes restoration after lesions^13–15^. Although some clinical trials using n-3 PUFA supplementation reported clinical benefits of PUFA, the number of patients in these trials was small or vitamin E was co-administered^16, 17^. Despite conflicting results, it is noteworthy that PUFA in PD need to be further investigated regarding their possible pathophysiological roles and nutraceutical potential.

Approximately 60% of the brain’s structural compounds are lipids, and PUFA are a major constituent, especially docosahexaenoic acid (DHA, C22:6n3) and arachidonic acid (AA, C20:4n6)^18, 19^. Humans can synthesize most fatty acids, except for α-linolenic acid (ALA, C18:3n3) and linoleic acid (LA, C18:2n6), which are the building blocks of n-3 and n-6 PUFA, respectively^20^. n-3 PUFA include eicosapentaenoic acid (EPA, C20:5n3) and DHA, which are synthesized from ALA through multiple reactions of desaturation, elongation, and β-oxidation. Derivatives of n-3 PUFA include prostaglandins, leukotrienes, resolvins, and protectin, which are classic anti-inflammatory lipid mediators^21^. On the other hand, n-6 PUFA include AA, which is synthesized from LA and further metabolized to prostaglandin and leukotriene, or potent mediators of thrombosis and pro-inflammatory mediators^22^. The endogenous synthetic process is not only energetically expensive but also insufficient to supply all cells with PUFA. Therefore, mammals complement PUFA via uptake from dietary sources. n-6 and n-3 fatty acids are not interconvertible due to lack of n-3 desaturase, are metabolically and functionally distinct, and often have opposite physiological effects. Therefore, dietary PUFA consumption and the intrinsic n-6/n-3 balance may play a role in physiological processes, including neuroinflammation, aging-related processes, and neurodegenerative diseases^23^.

Previously, either a food questionnaire or direct measurement of PUFA was conducted to investigate the role of PUFA in PD, which was insufficient to reveal the association between long-term dietary habits and plasma PUFA levels. In this study, we simultaneously examined the dietary fat intake and plasma concentrations of PUFA as an explorative study to identify relevant dietary or clinical factors associated with plasma PUFA levels.

## Results

**Figure 1:**
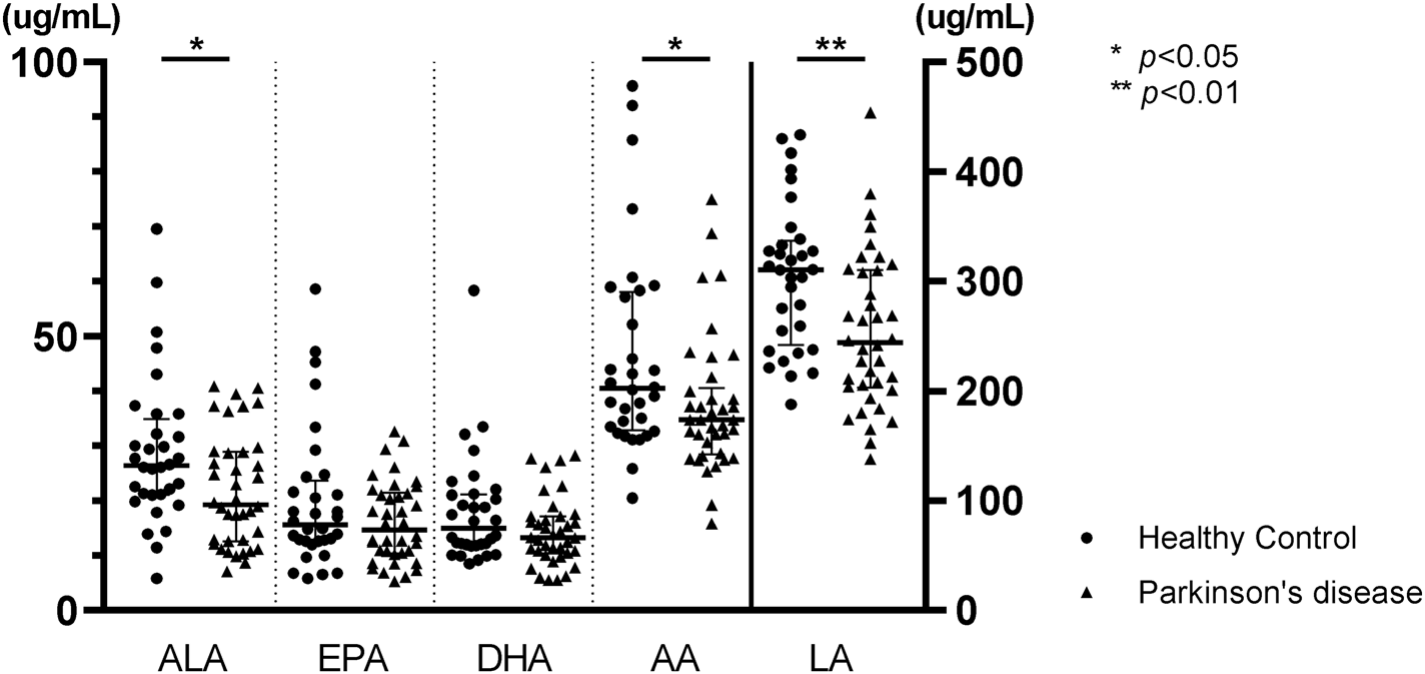
Plasma polyunsaturated fatty acid concentrations in Parkinson’s disease. *ALA *alpha-linolenic acid, *EPA* eicosapentaenoic acid, *DHA* docosahexaenoic acid, *LA* linoleic acid, *AA* arachidonic acid.

Demographic factors, co-morbidity, and other lipid levels were previously described^24^ and did not differ between patients with PD and controls. In contrast, the Wexner constipation score and Korean version of the Non-Motor Symptom Scale (K-NMSS) scores were significantly higher in patients with PD than in controls (Table 1). Although the difference was not statistically significant, the proportion of men and participants with diabetes mellitus (DM) was higher in the PD group than in the control group (45% versus 30% and 21% versus 9%, respectively). The plasma levels of ALA, LA, and AA were significantly lower in patients with PD than in controls, although individual values overlapped between the two groups (*p* = 0.017, *p* = 0.003, and *p* = 0.024, respectively; Table 1; Fig. 1). The ratio of n-6 to n-3 PUFA (n-6/n-3 ratio) from the five PUFA and the plasma levels of EPA and DHA were not different between patients with PD and controls. The effects of group on plasma levels of ALA, LA, and AA were maintained after controlling for age and sex by analysis of covariance [F(1,66) = 5.045, *p* = 0.028 for ALA, F(1,66) = 9.059, *p* = 0.004, and F(1,66) = 5.431, *p* = 0.023 for AA; Table 2]. When subgroup analysis was performed to evaluate the effect of anti-parkinsonian medications, only the plasma level of LA was lower in patients taking catechol-*O*-methyltransferase inhibitor compared to those who did not (211.8 ± 50.4 μg/mL versus 269.2 ± 71.0 μg/mL, respectively, *p* = 0.025 [Supplementary Table S1]).

**Table 1:** Demographic and clinical data of the study participants and their plasma lipid levels.

	PD (n = 38)	HC (n = 33)	*p* value
Age, years	66.0 (5.7)	66.1 (5.8)	0.945
Male, n (%)	17 (44.7)	10 (30.3)	0.211^a^
Hypertension, n (%)	12 (31.6)	12 (36.4)	0.802^a^
Diabetes mellitus, n (%)	8 (21.1)	3 (9.1)	0.202^a^
BMI, kg/m^2^	23.8 (2.8)	23.1 (2.7)	0.253^b^
WCS (0–30)	3.2 (3.7)	0.1 (0.5)	**< 0.001**
K-NMSS (0–360)	8.6 (6.5)	3.0 (2.9)	**< 0.001**
Disease duration, years	4.2 (2.7)	–	
UPDRS I	2.1 (2.1)	–	
UPDRS II	6.2 (3.9)	–	
UPDRS III	19.8 (7.9)	–	
Hoehn and Yahr stage	2.0 (0.6)	–	
LEDD, mg/d	607.7 (331.2)	–	
Total cholesterol, mg/dL	189.2 (33.2)	203.9 (41.5)	0.127
TG, mg/dL	123.2 (67.5)	135.4 (59.2)	0.278
LDL, mg/dL	123.5 (26.2)	132.8 (29.0)	0.160^b^
HDL, mg/dL	52.1 (10.3)	51.8 (15.9)	0.858
ALA, µg/mL	21.8 (1.7)	29.0 (2.4)	**0.017**
EPA, µg/mL	16.1 (1.2)	19.8 (2.3)	0.396
DHA, µg/mL	14.4 (1.0)	18.0 (1.8)	0.114
LA, µg/mL	254.1 (70.4)	305.6 (66.4)	**0.003**^**b**^
AA, µg/mL	37.3 (2.0)	46.4 (3.3)	**0.024**
n-6/n-3 ratio^c^	6.4 (2.5)	6.4 (1.7)	0.163^b^

Data are described as mean (standard deviation) or frequency. The *p* values marked in bold indicate statistically significant differences between the two groups.

For group comparisons, the Mann–Whitney *U* test was used if not otherwise indicated.

^a^Pearson’s chi-squared test; ^b^Student’s *t* test; ^c^n-6/n-3 ratio = (LA + AA)/(ALA + EPA + DHA).

*PD *Parkinson’s disease, *HC* healthy control, *BMI* body mass index, *WCS* Wexner constipation score, *K-NMSS* Korean Non-Motor Symptoms Scale, *UPDRS* Unified Parkinson’s Disease Rating Scale, *LEDD* levodopa-equivalent daily dose, *TG* triglyceride, *LDL* low-density lipoprotein, *HDL* high-density lipoprotein, *ALA* alpha-linolenic acid, *EPA* eicosapentaenoic acid, *DHA* docosahexaenoic acid, *LA* linoleic acid, *AA* arachidonic acid.

**Table 2:** Results of ANCOVA for individual PUFA after controlling for age and sex.

PUFA	Factor	dF	F	*p* value
ALA	**Group (PD)**	**1**	**5.045**	**0.028**
	Sex (male)	1	2.961	0.090
	Age	1	1.883	0.175
	Error	66		
EPA	Group (PD)	1	1.915	0.171
	Sex (male)	1	0.309	0.580
	Age	1	2.468	0.121
	Error	66		
DHA	Group (PD)	1	3.352	0.072
	Sex (male)	1	0.046	0.831
	Age	1	0.818	0.369
	Error	66		
LA	**Group (PD)**	**1**	**9.059**	**0.004**
	Sex (male)	1	0.056	0.814
	Age	1	0.632	0.429
	Error	66		
AA	**Group (PD)**	**1**	**5.431**	**0.023**
	Sex (male)	1	0.180	0.673
	Age	1	0.432	0.513
	Error	66		
n-6/n-3 ratio^a^	Group (PD)	1	0.132	0.717
	Sex (male)	1	1.560	0.216
	Age	1	2.287	0.135
	Error	66		

The values marked in bold indicate statistically significant differences.

*ANCOVA *analysis of covariance, *PUFA* polyunsaturated fatty acid, *ALA* alpha-linolenic acid, *EPA* eicosapentaenoic acid, *DHA* docosahexaenoic acid, *LA* linoleic acid, *AA* arachidonic acid.

^a^n-6/n-3 ratio = (LA + AA)/(ALA + EPA + DHA).

**Figure 2:**
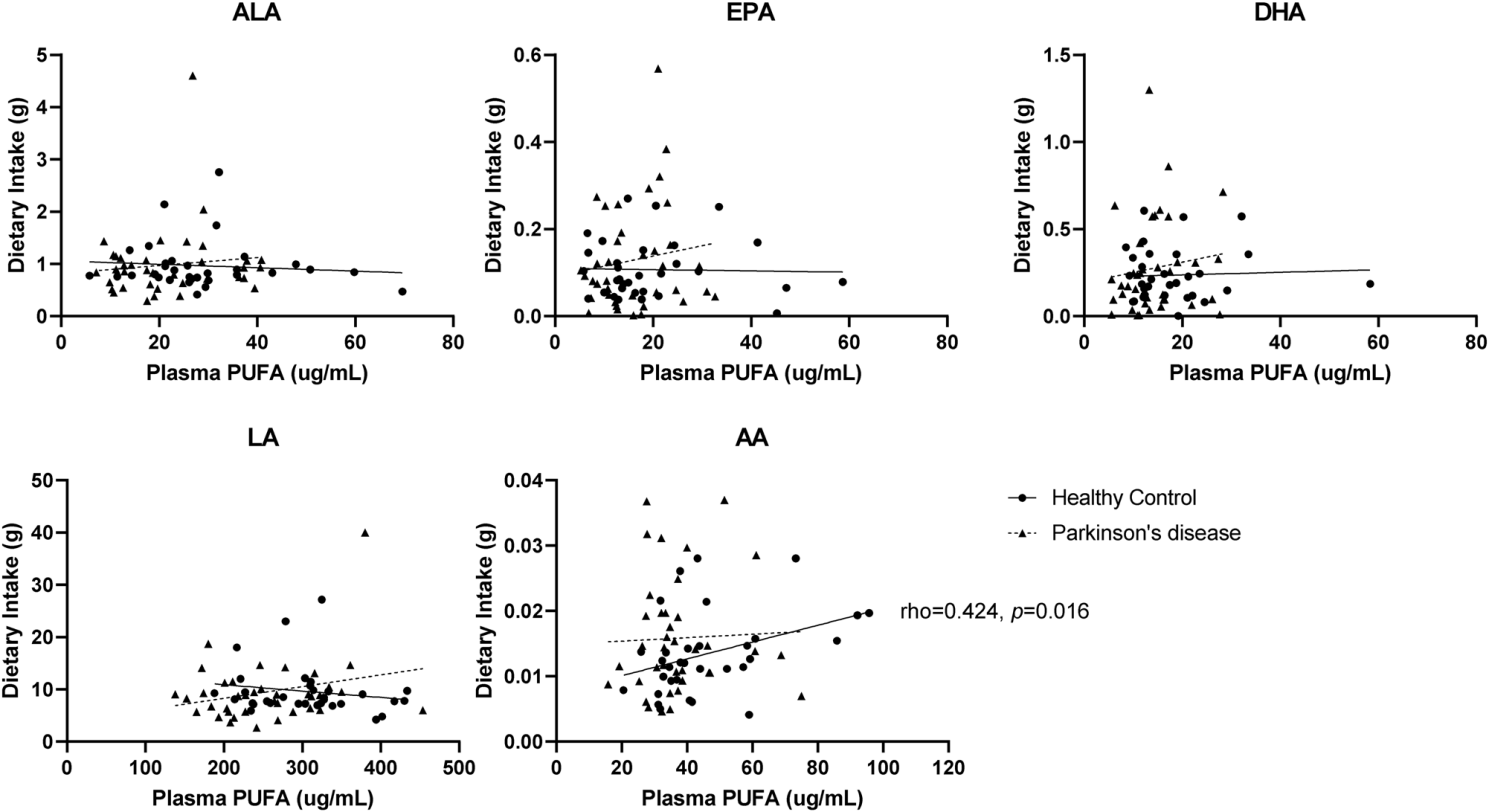
No associations between dietary intake and plasma concentration of polyunsaturated fatty acids in Parkinson’s disease. *rho* = Spearman’s correlation coefficients. *PUFA* polyunsaturated fatty acids, *ALA* alpha-linolenic acid, *EPA* eicosapentaenoic acid, *DHA* docosahexaenoic acid, *LA* linoleic acid, *AA* arachidonic acid.

There were no differences in dietary intake including total energy, total fat, cholesterol, total fatty acids, saturated fatty acids, monounsaturated fatty acids, and PUFA between patients with PD and controls (Table 3). The n-6/n-3 ratio based on the dietary intake of the five PUFA was not different between the two groups. There were no significant associations between dietary intake and plasma PUFA levels in patients with PD (Fig. 2). In the control group, only AA intake correlated with plasma concentration (ρ = 0.424, *p* = 0.016).

**Table 3:** Dietary intake based on a food frequency questionnaire in PD.

g	PD (n = 38)	HC (n = 33)	*p* value
Total energy intake, kcal	1935.05 (634.77)	1938.19 (493.88)	0.917
**Fat, g**	49.78 (26.48)	46.72 (16.57)	0.963
Vegetable fat	25.95 (16.08)	26.74 (13.67)	0.572
Animal fat	23.83 (12.82)	19.98 (6.03)	0.105^a^
Cholesterol, mg	343.82 (177.78)	358.11 (151.97)	0.489
Total fatty acids, g	34.09 (18.73)	31.58 (11.71)	1.000
Saturated fatty acids	12.57 (7.55)	10.51(3.39)	0.135^a^
MUFA	14.82 (8.95)	13.49 (4.52)	0.963
PUFA	11.56 (7.81)	11.42 (5.17)	0.704
ALA (C18:3n3)	0.98 (0.70)	0.96 (0.46)	0.908
EPA (C20:5n3)	0.13 (0.12)	0.11 (0.07)	0.881
DHA (C22:6n3)	0.28 (0.28)	0.24 (0.16)	0.721
LA (C18:2n6)	9.51 (6.17)	9.56 (4.73)	0.747
AA (C20:4n6)	0.02 (0.01)	0.01 (0.01)	0.475
n-6/n-3 ratio^b^	7.30 (1.65)	7.40 (1.57)	0.799^a^

Data are described as mean (standard deviation).

For group comparisons, the Mann–Whitney *U* test was used if not otherwise indicated.

*PD *Parkinson’s disease, *HC* healthy control, *MUFA* monounsaturated fatty acid, *PUFA* polyunsaturated fatty acid, *ALA* alpha-linolenic acid, *EPA* eicosapentaenoic acid, *DHA* docosahexaenoic acid, *LA* linoleic acid, *AA* arachidonic acid.

^a^Student’s *t* test.

^b^n-6/n-3 ratio = (LA + AA)/(ALA + EPA + DHA).

The severity of motor symptoms (UPDRS III) was negatively associated with plasma ALA and LA concentrations after adjusting for age and sex (ρ = − 0.351, *p* = 0.033 for ALA and ρ = − 0.424, *p* = 0.009 for LA after adjusting for age; ρ = − 0.349, *p* = 0.034 for ALA and ρ = − 0.424, *p* = 0.009 for LA after adjusting for sex; Table 4). Plasma DHA and AA levels were positively associated with non-motor symptom severity (UPDRS I; ρ = 0.355, *p* = 0.031 for DHA and ρ = 0.362, *p* = 0.028 for AA after adjusting for age; ρ = 0.441, *p* = 0.006 for DHA and ρ = 0.481, *p* = 0.003 for AA after adjusting for sex; Table 4). The n-6/n-3 ratio was negatively associated with non-motor symptom scales (K-NMSS; ρ = − 0.358, *p* = 0.029 after adjustment for age; ρ = − 0.351, *p* = 0.033 after adjusting for sex; Table 4). No associations were found between plasma levels of PUFA and disease duration, constipation, UPDRS II, Hoehn and Yahr stage, or total anti-parkinsonian medication. Supplementary Table S2 shows the results of the partial correlation analysis between the scores of the UPDRS items and the plasma concentrations of PUFA.

**Table 4:** Association between clinical parameters and plasma levels of PUFA in PD.

	ALA		EPA		DHA		LA		AA		n-6/n-3 ratio^a^	
	ρ	*P*	ρ	*p*	ρ	*p*	ρ	*p*	ρ	*p*	ρ	*p*
**Age**^**b**^												
Disease duration, years	− 0.290	0.082	− 0.030	0.861	− 0.007	0.969	− 0.116	0.493	0.019	0.910	0.159	0.349
WCS	− 0.101	0.552	0.001	0.996	0.008	0.963	0.184	0.276	0.183	0.279	0.165	0.329
K-NMSS	− 0.210	0.211	− 0.227	0.176	− 0.104	0.540	− 0.173	0.306	0.211	0.210	**− 0.358**	**0.029**
UPDRS I	0.069	0.687	0.207	0.220	**0.355**	**0.031**	− 0.203	0.229	**0.362**	**0.028**	− 0.231	0.170
UPDRS II	− 0.056	0.742	0.198	0.241	0.258	0.123	− 0.199	0.236	0.087	0.609	− 0.025	0.886
UPDRS III	**− 0.351**	**0.033**	− 0.185	0.272	− 0.062	0.716	**− 0.424**	**0.009**	− 0.184	0.275	− 0.149	0.380
H&Y stage	− 0.058	0.731	0.113	0.504	0.108	0.524	− 0.038	0.824	0.183	0.279	0.053	0.757
LEDD, mg/d	− 0.187	0.269	0.005	0.978	0.172	0.308	− 0.068	0.690	0.036	0.831	0.159	0.349
**Sex**^**c**^												
Disease duration, years	− 0.273	0.102	− 0.012	0.945	0.009	0.958	− 0.110	0.518	0.034	0.840	0.135	0.424
WCS	− 0.103	0.546	0.003	0.985	− 0.031	0.857	0.161	0.342	0.089	0.600	0.155	0.358
K-NMSS	− 0.191	0.257	− 0.215	0.202	− 0.052	0.761	− 0.147	0.384	0.267	0.110	**− 0.351**	**0.033**
UPDRS I	0.101	0.552	0.221	0.189	**0.441**	**0.006**	− 0.147	0.386	**0.481**	**0.003**	− 0.244	0.145
UPDRS II	− 0.044	0.794	0.209	0.215	0.263	0.116	− 0.194	0.251	0.089	0.601	− 0.048	0.777
UPDRS III	**− 0.349**	**0.034**	− 0.176	0.299	− 0.066	0.699	**− 0.424**	**0.009**	− 0.178	0.293	− 0.108	0.524
H&Y stage	− 0.134	0.427	0.082	0.628	0.065	0.700	− 0.057	0.739	0.106	0.534	0.012	0.943
LEDD, mg/d	− 0.133	0.431	0.039	0.819	0.220	0.191	− 0.047	0.784	0.100	0.555	0.135	0.424

Data are described as mean (standard deviation); the correlation coefficients and *p* values marked in bold indicate statistically significant differences.

Spearman’s partial correlation analysis was applied after adjusting for age^b^ and sex^c^.

*PUFA *polyunsaturated fatty acids, *PD* Parkinson’s disease, *ALA* alpha-linolenic acid, *EPA* eicosapentaenoic acid, *DHA* docosahexaenoic acid, *LA* linoleic acid, *AA* arachidonic acid, *WCS* Wexner constipation score, *K-NMSS* Korean Non-Motor Symptoms Scale, *UPDRS* Unified Parkinson’s Disease Rating Scale, *H&Y stage* Hoehn and Yahr stage, *LEDD* levodopa equivalent daily dose.

^a^n-6/n-3 ratio = (LA + AA)/(ALA + EPA + DHA).

## Discussion

In this study, we compared the dietary intake and plasma levels of two essential fatty acids and their long chain metabolites in patients with PD and controls. In patients with PD, plasma ALA levels were lower among the three n-3 PUFA examined, and both n-6 PUFA, LA, and AA, showed lower levels compared to controls. However, the reported dietary consumption of PUFA in patients with PD was similar to that in controls, and plasma levels were not correlated with dietary intake in PD. Lower plasma levels of ALA and LA were associated with more severe motor symptoms in patients with PD, while higher levels of plasma DHA and AA were associated with more severe non-motor symptoms in PD, suggesting that fatty acid metabolism is altered in PD.

Previous epidemiological studies have shown conflicting results on the role of PUFA in the risk of PD^4, 8–10, 25^. A recent review summarized that the risk of PD is lower in people with higher intake of n-3 PUFA (ALA) and the n-3/n-6 ratio, while it was higher in those with higher intake of n-6 PUFA (AA)^26^. In our study, dietary history of PUFA intake and calculated n-6/n-3 ratio were not different between patients with PD and controls. Although we did not assess the risk of PD associated with PUFA, our findings are inconsistent with those of previous diet studies. One possible explanation for these inconsistencies is that we recruited patients with an established diagnosis of PD. Considering the mean disease duration of 4 years or more, food consumption behavior could have been modified by consumption of “healthier” foods after the diagnosis, thereby blunting unhealthy patterns during the preclinical or early stages of the disease. Food behavior differences were revealed in a study showing that a larger proportion of patients with PD (76%) in South Korea took complementary or alternative medicine than in Western countries^27, 28^. Changes in the pattern of food intake before and after the diagnosis of PD have not been fully studied, except for the increased total amount of energy intake after PD diagnosis^29^. Further studies are required to assess the influence of PD diagnosis on changes in the pattern of food intake.

Only a couple of studies directly measured plasma PUFA levels, in which both n-3 and n-6 PUFA levels were lower in patients with PD while the levels of other lipids were not different between PD patients and controls^11, 12^. These results are consistent with the results of our study. However, previous studies have reported decreased PUFA levels through profiling of serum metabolites without information on the diet. The discrepancy between dietary intake and plasma concentrations of PUFA was first reported in patients with PD. When assessing the association between dietary intake and plasma PUFA in PD, potential factors could include endogenous synthesis, enteric absorption, and utilization of PUFA. An early hypothesis has been that neuroinflammation could contribute to the pathophysiological etiology of PD^2^. Aberrant neuroinflammatory activation might reduce n-3 and n-6 PUFA levels by disrupting the homeostasis of both anti- and pro-inflammatory signals and by promoting compensatory or pathological metabolic pathways of fatty acids. Second, PUFA are absorbed in the small intestine. Thus, small intestinal bacterial overgrowth can hinder PUFA absorptions^30, 31^. Small intestinal bacterial overgrowth is frequently reported in patients with PD and has negative effects on clinical features^32^. Inflammation or disturbed osmolarity by bacteria may hamper normal absorption^30, 32^. In this study, no difference in dietary PUFA intake between patients with PD and controls was observed, and the positive association between dietary intake and plasma levels of AA in the control group suggested that malabsorption was the cause of decreased PUFA levels in PD. Third, PUFA are utilized by neurons, and expedited utilization may be associated with pathologic changes in the local milieu, such as oxidative stress in PD. Fatty acid-binding protein (FABP) translocates PUFA from the extracellular space into neurons. In PD, FABP expression is elevated in the substantia nigra (SN)^33^. As PUFA protect against oxidative stress, this upregulation may protect the SN which is very energy-demanding and vulnerable to oxidative stress.

In terms of local dietary characteristics, it might be difficult to generalize the results from plasma n-3 PUFA levels with high intake of EPA and DHA in South Korea^34^, since blood levels of EPA and DHA have been reported to be associated with the reported fish intake or supplementation in the general population^35^. The exclusive dietary sources of EPA and DHA are seafood, including oily fish and shellfish. The global mean intake of seafood n-3 PUFA is 163 mg/day, while South Korea ranks as one of the regions with the highest mean intake (708 mg/day)^36^. The mean consumption in the United States is 141 mg/day^36^. These cultural dietary differences can influence plasma PUFA concentrations, along with several known variable factors. For example, it is unknown how much intake of each n-3 PUFA quantitatively affects the plasma concentrations and the capacity of humans to synthesize EPA and DHA from ALA is variable. Additionally, patients with PD might have dysregulated PUFA homeostasis. Serum levels of n-3 PUFA in South Korea (8.62 ± 0.14%) are significantly higher than those in the US (4.18 ± 0.14%)^37^, and the plasma levels of PUFA in the high-set control group can blunt the subtle difference between PD and control groups.

In clinical analyses, the plasma levels of PUFA were negatively associated with the severity of motor symptoms, implying a beneficial effect of PUFA. Previous studies using n-3 PUFA produced inconsistent results, whereas clinical studies using n-6 PUFA were not conducted because of the unfavorable results obtained regarding the association between n-6 PUFA and PD risk^9, 10, 26^. In our study, both n-3 (ALA) and n-6 (LA) PUFA were negatively associated with parkinsonian motor symptoms, while other n-3 (DHA) and n-6 (AA) PUFA had a positive association with the severity of non-motor symptoms. Therefore, the clinical effects of PUFA in PD are not straightforward.

Previous studies have reported an abnormal PUFA composition in the brains of patients with PD compared to controls. In the SN, PUFA levels are decreased along with increased malondialdehyde levels, an intermediate product of lipid peroxidation, which suggests that parkinsonian nigral dopamine neurons might be exposed to excessive oxidative damage and are susceptible to lipid peroxidation^6, 38^. Other studies revealed that the highly peroxidable DHA is increased in the frontal cortices and amygdala but decreased in the SN of patients with Lewy body disease compared to age-matched controls^39^. On the other hand, purified lipid rafts from patients with early-stage or incidental PD exhibit reduced DHA and AA levels in the frontal cortices^40^. These results suggest that aberrant compositions of peroxidable PUFA in the brain might indicate their disease-specific roles in PD, accompanied by dysregulation and remodeling of lipid metabolism. Furthermore, levodopa-treated patients with PD showed cortical PUFA compositions similar to those of the matched controls, while patients with PD with levodopa-induced dyskinesia showed higher cortical levels of AA compared to controls and patients with PD without motor complications^41^. Levodopa administration in primate models of PD increases AA levels and decreases DHA concentrations in the cortex compared to drug-naïve ones. Therefore, marked changes in PUFA composition might be responsible for the pathophysiology of PD and disease progression.

There are several limitations of this study to be considered. First, because of the small sample size and high variability, the results need to be interpreted carefully, albeit we attempted to adjust for all possible confounders. Therefore, low serum levels of some essential PUFA in patients with PD should be validated in future larger confirmatory studies. Second, subjective dietary assessment methods use a retrospective closed-ended questionnaire, which can be affected by recall bias. However, the food frequency questionnaire (FFQ) has been developed using actual dietary data collected by open-ended surveys, is widely used in epidemiologic studies, and was modified according to the Korean diet and recipes^42^. Third, since PD is associated with a higher risk of dementia, recall ability could be lower in patients with PD^43^. However, we excluded dementia based on the diagnostic criteria of PD, and only one patient reported subjective cognitive impairment (score = 2) in UPDRS Part I. Fourth, the association of plasma PUFA levels with PD could be afflicted by the potential for reverse causality in the observational study. In other words, the lower plasma levels of PUFA could be the results of PD-specific pathophysiological changes or they might be a risk factor of PD. Finally, one limitation is related to the reliability of a single cross-sectional measurement of PUFA levels. In this study, to prevent possible dietary effects, we asked the participants to fast for more than 12 h before blood sampling. We simultaneously measured the levels of non-PUFA lipids to ensure the reliability of PUFA measurements, which were not different between patients with PD and controls. Although we did not repeat the PUFA measurement, repeated measurements of PUFA levels over 2–3 years showed consistent results in women from Nurses’ Health Studies^44^.

In conclusion, the simultaneous assessment of food intake history and plasma levels of PUFA was first performed in patients with PD compared to controls. Overconsumption of PUFA by vulnerable neurons with both beneficial and detrimental effects may explain the lower plasma levels of ALA, LA, and AA in PD without differences in dietary consumption. Conflicting associations between PUFA and clinical severity raise questions about the benefits of PUFA supplementation in PD.

## Methods

### Subjects

Thirty-eight patients with PD and 33 controls aged between 50 and 75 years were included. Patients were diagnosed with PD according to the United Kingdom Parkinson’s Disease Society Brain Bank Clinical Diagnosis Criteria^45^. The inclusion criteria were as follows: patients not receiving antibiotics, immune-related drugs, and vaccines (for 3 months), lipid-lowering drugs (for 1 month), and vitamin supplements, n-3 fatty acids, prebiotics, and probiotics (for 2 weeks)^24^. The exclusion criteria were as follows: patients with secondary parkinsonism, other significant central nervous system diseases, malignancy within the last 3 years, and significant gastrointestinal disorders^24^. Healthy control subjects were recruited from volunteers via posters on notice boards in the hospital and the patients’ spouses. All control subjects fulfilled the above inclusion and exclusion criteria except for those regarding PD and were confirmed to have no abnormal signs on neurological examinations. The study was approved by the local ethics committee (Institutional Review Boards of Kyung Hee University Hospital, # KHUH 2017‐08‐035) and all subjects provided written informed consent before inclusion in the study in accordance with the Code of Ethics of the World Medical Association (Declaration of Helsinki).

### Clinical and nutritional evaluation

All subjects were interviewed by a trained interviewer using a structured questionnaire to obtain information on demographics, height, weight, and medical history (disease duration and medications). We used a validated 122-item semiquantitative food frequency questionnaire (FFQ) to assess usual dietary intake over the past 12 months. The items were selected based on commonly consumed foods using the Korea National Health and Nutrition Examination Survey^46, 47^. A skilled nutritionist interviewed all participants for more than 30 min about how often and how much food they ate. The dietary intake of each nutrient was calculated based on the subjects’ responses to the FFQ and the Korean Food Composition Table by the Korean Nutrition Society program CAN-Pro 4.0 (Nutritional Assessment Program 2011 Korean Nutrition Society, Seoul, Korea). The Unified Parkinson’s Disease Rating Scale (UPDRS), Wexner constipation score, and K-NMSS were used for clinical evaluation of the subjects. Each patient was assessed in "medication-off" state or at least after 12 h after taking the last dose of anti-parkinsonian medications. The levodopa-equivalent daily dose was calculated based on a previous reference^48^.

### Sample collection and plasma lipid analysis

Blood samples were collected in the morning after the subjects were required to fast for at least 12 h. We measured the levels of cholesterol, triglycerides, high- and low-density lipoprotein, and PUFA, including ALA, EPA, DHA, LA, and AA.

All blood samples were prepared in a non-additive blood collecting tube by adding 1 mg/mL ethylenediaminetetraacetic acid and 1 mg/mL reduced glutathione. Plasma was immediately separated by centrifugation at 3000 rpm for 10 min. The plasma was treated with butylated hydroxytoluene-methanol (10 µL/mL) and stored at − 80 °C.

For PUFA analysis, the samples were dissolved in methanol containing methyl heptadecanoate (3 g/L) as an internal standard. Acetyl chloride was added to the dissolved samples for acid-catalyzed esterification and transesterification and incubated for 45 min. For neutralization, potassium carbonate solution (6% in water) was mixed gently with hexane and subsequently placed on ice for 30 min. After cooling, the tubes were centrifuged for 10 min at 3000 rpm. The extracted lipids from the supernatant were dried under nitrogen gas and washed with hexane using a solvent.

The solution was injected in pulsed-split mode into an Agilent 7890 B gas chromatograph with a flame ionization detector using an Agilent DB-WAX capillary column (30 m × 0.25 μm × 0.25 μm). The inlet and detector temperatures were 250 °C and 270 °C, respectively, and nitrogen was used as the carrier gas at a rate of 1 mL/min. The concentration of long-chain fatty acids was calculated using the peak height ratio method^49^.

### Data and statistical analyses

We applied Student’s *t *test and Mann–Whitney *U* test to compare the means of two independent continuous variables if data showed a normal distribution. Categorical data were compared using Pearson’s chi-squared test. Partial Spearman’s rank correlation coefficient analysis was applied to determine bivariate correlations after adjusting for age and sex. Analysis of covariance for each PUFA was performed to correct for potential confounding variables, with age and sex as covariates. The Statistical Package for the Social Sciences (SPSS) software (version 25.0; SPSS Inc., Chicago, IL, USA) was used for statistical analysis, with *p* < 0.05, considered statistically significant (two-tailed).

## Supplementary Information

Supplementary Information 1.

## Data Availability

The datasets collected and analyzed for the current study are available from the corresponding author upon reasonable request.

## Abbreviations

PD: Parkinson’s disease
PUFA: Polyunsaturated fatty acids
ALA (C18:3N3): Alpha-linolenic acid
EPA (C20:5N3): Eicosapentaenoic acid
DHA (C22:6N3): Docosahexaenoic acid
LA (C18:2N6): Linoleic acid
AA (C20:4N6): Arachidonic acid
FFQ: Food frequency questionnaire
UPDRS: Unified Parkinson’s Disease Rating Scale
WCS: Wexner constipation score
K-NMSS: Korean version of the Non-Motor Symptom Scale
