# Supplementary material for: Dietary intake and plasma levels of polyunsaturated fatty acids in early-stage Parkinson’s disease
Source: Sci Rep. 2021 Jun 14;11:12489. doi: 10.1038/s41598-021-92029-x (PMC8203700; doi:10.1038/s41598-021-92029-x)
Supplement: Supplementary file 1 — Supplementary Information 1. [file 41598_2021_92029_MOESM1_ESM.pdf]

Dietary Intake and Plasma Levels of Polyunsaturated Fatty Acids in Early-Stage Parkinson's Disease

Dallah Yoo, MD[1], Yunsook Lim, PhD[2], Yiseul Son, BSc[2], Hyunkyung Rho, MEd[2], Chaewon Shin, MD, PhD[3], Tae-Beom Ahn, MD, PhD[1]*

[1]Department of Neurology, Kyung Hee University Hospital, Kyung Hee University College of Medicine, Seoul, Republic of Korea

[2]Department of Food and Nutrition College of Human Ecology, Kyung Hee University, Seoul, Republic of Korea

[3]Department of Neurology, Chungnam National University Hospital, College of Medicine, Chungnam National University, Daejeon, Korea

Correspondence to:

Tae-Beom Ahn, MD, PhD

Department of Neurology, Kyung Hee University Hospital

23, Kyungheedae-ro, Dongdaemun-gu, Seoul, Republic of Korea (02447)

Tel: +82-2-958-8448, Fax: +82-2-958-8490, E-mail address: taebeom.ahn@khu.ac.kr

Supplementary Table S1. Plasma polyunsaturated fatty acid levels according to medication in Parkinson's disease

Plasma level (µg/mL)	Levodopa			MAO-B inhibitor			Dopamine agonist			COMT inhibitor			Amantadine		
	Yes n=28	No n=10	*p*-value	Yes n=28	No n=10	*p*-value	Yes n=26	No n=12	*p*-value	Yes n=10	No n=28	*p*-value	Yes n=18	No n=20	*p*-value
**ALA**	21.1 (9.7)	23.8 (11.9)	0.491	21.4 (10.3)	23.0 (10.6)	0.690	21.9 (10.1)	21.8 (11.0)	0.985	18.3 (10.1)	23.1 (10.2)	0.286[a]	20.7 (9.8)	22.9 (10.8)	0.522
**EPA**	16.8 (7.7)	14.1 (6.0)	0.328	15.1 (7.0)	19.0 (7.7)	0.141	16.4 (7.4)	15.5 (7.4)	0.841[a]	15.1 (7.0)	16.5 (7.5)	0.604	16.2 (7.1)	16.0 (7.7)	0.951
**DHA**	14.8 (6.1)	13.1 (6.5)	0.465	14.0 (6.2)	15.3 (6.2)	0.584	14.6 (6.7)	13.8 (5.0)	0.724	16.0 (7.4)	13.8 (5.6)	0.321	14.0 (6.9)	14.6 (5.5)	0.443[a]
**LA**	246.6 (67.5)	275.1 (77.5)	0.277	259.6 (76.1)	238.7 (51.4)	0.427	261.9 (78.9)	237.1 (45.4)	0.319	**211.8 (50.4)**	**269.2 (71.0)**	**0.025**	247.5 (74.3)	260.0 968.1)	0.593
**AA**	38.2 (14.0)	34.9 (7.2)	0.757[a]	38.8 (13.8)	33.2 (7.3)	0.442[a]	38.6 (13.2)	34.6 (11.1)	0.312[a]	38.9 (17.8)	36.8 (10.5)	0.568[a]	37.9 (15.8)	36.9 (9.2)	0.828[a]

Data are described as mean (standard deviation); The *p*-values marked in bold indicate significant differences between groups.

For group comparisons, Student's *t*-test was used if not otherwise indicated; [a]Mann-Whitney *U* test

Abbreviations: PUFAs, polyunsaturated fatty acids; MAO-B, monoamine oxidase-B; COMT, catechol-O-methyltransferase; ALA, alpha-linolenic acid; EPA, eicosapentaenoic acid; DHA, docosahexaenoic acid; LA, linoleic acid; AA, arachidonic acid

Supplementary Table S2. Association between the Unified Parkinson's Disease Rating Scale item scores and plasma polyunsaturated fatty acid levels in Parkinson's disease

Age-adjusted	Items	Plasma concentrations (μg/mL)											
		ALA		EPA		DHA		LA		AA		ω-6/ω-3 ratio[a]	
		ρ	*p*	ρ	*p*	ρ	*p*	ρ	*p*	ρ	*p*	ρ	*p*
**UPDRS I**	**Intellectual impairment**	0.069	0.687	0.207	0.220	**0.355**	**0.031**	-0.203	0.229	**0.362**	**0.028**	-0.358	0.029
	Thought disorder	-0.056	0.742	0.198	0.241	0.258	0.123	-0.199	0.236	0.087	0.609	-0.231	0.170
	**Depression**	**-0.351**	**0.033**	-0.185	0.272	-0.062	0.716	**-0.424**	**0.009**	-0.184	0.275	-0.025	0.886
	**Motivation/initiative**	-0.198	0.240	-0.101	0.551	-0.033	0.844	-0.140	0.407	**0.332**	**0.044**	-0.059	0.731
**UPDRS II**	Speech	0.093	0.585	-0.134	0.429	-0.154	0.363	0.117	0.491	0.003	0.987	0.015	0.929
	**Salivation**	0.055	0.745	0.142	0.403	0.297	0.075	-0.216	0.198	**0.442**	**0.006**	-0.318	0.055
	**Swallowing**	-0.111	0.513	-0.086	0.615	0.267	0.110	**-0.414**	**0.011**	0.298	0.073	-0.272	0.103
	**Handwriting**	0.094	0.581	0.278	0.095	0.209	0.214	-0.049	0.771	0.262	0.118	**-0.330**	**0.046**
	Cutting food and handling utensils	-0.101	0.553	0.095	0.576	0.084	0.623	-0.144	0.396	-0.061	0.722	0.008	0.965
	Dressing	0.138	0.415	-0.015	0.928	-0.184	0.276	0.134	0.429	0.047	0.782	0.121	0.475
	Hygiene	-0.052	0.760	0.187	0.268	0.052	0.758	-0.118	0.488	0.127	0.455	-0.134	0.428
	Turning in bed and adjusting bed cloths	-0.242	0.148	-0.262	0.117	-0.216	0.199	-0.089	0.600	0.005	0.976	0.140	0.410

	Falling (unrelated to freezing)	0.126	0.458	0.264	0.115	0.186	0.271	-0.052	0.762	0.064	0.708	-0.270	0.106
	Freezing when walking	0.143	0.400	0.190	0.260	0.178	0.293	0.004	0.981	0.030	0.859	-0.246	0.141
	Walking	-0.186	0.271	-0.156	0.356	-0.080	0.639	-0.306	0.066	-0.060	0.726	0.037	0.827
	Tremor	-0.143	0.397	0.166	0.325	0.072	0.672	-0.184	0.276	-0.068	0.691	-0.081	0.632
	Sensory complaints related to parkinsonism	0.040	0.815	0.171	0.311	0.173	0.307	0.046	0.786	0.023	0.890	-0.111	0.514
**UPDRS III**	Speech	0.014	0.936	0.196	0.246	0.127	0.453	-0.317	0.056	0.029	0.863	-0.281	0.092
	Facial expression	-0.027	0.872	-0.202	0.230	-0.039	0.818	-0.103	0.543	-0.177	0.295	0.073	0.669
	Tremor at rest	0.059	0.728	-0.050	0.769	0.056	0.744	-0.129	0.447	0.143	0.399	-0.199	0.238
	Action or postural tremor of hands	-0.196	0.244	-0.005	0.975	-0.155	0.360	-0.267	0.110	-0.165	0.328	0.022	0.898
	**Rigidity**	**-0.331**	**0.046**	-0.249	0.137	-0.070	0.679	**-0.380**	**0.020**	-0.202	0.231	0.005	0.979
	**Finger taps**	-0.179	0.289	-0.292	0.080	-0.237	0.158	-0.134	0.431	**-0.362**	**0.028**	0.094	0.580
	Hand movements	-0.232	0.167	-0.147	0.386	0.001	0.994	-0.096	0.570	-0.042	0.804	0.040	0.814
	Rapid alternating movements of hands	-0.084	0.620	0.009	0.957	0.055	0.748	-0.149	0.379	-0.301	0.070	-0.123	0.469
	Leg agility	-0.133	0.432	0.053	0.753	0.203	0.227	-0.158	0.350	0.277	0.097	-0.147	0.387
	**Arising from chair**	**-0.352**	**0.033**	-0.216	0.200	-0.031	0.855	**-0.421**	**0.010**	-0.061	0.719	0.090	0.595
	Posture	-0.038	0.823	0.112	0.511	0.028	0.868	-0.191	0.258	0.110	0.515	-0.200	0.236
	**Gait**	-0.190	0.261	-0.169	0.318	-0.041	0.808	**-0.417**	**0.010**	-0.073	0.667	0.031	0.854

	Postural stability	-0.086	0.612	0.064	0.705	0.049	0.773	-0.237	0.159	0.011	0.948	-0.076	0.655
	Body bradykinesia and hypokinesia	-0.230	0.171	-0.030	0.860	0.019	0.910	-0.156	0.357	-0.109	0.522	0.044	0.794

**Sex-adjusted**	**Items**	Plasma concentrations (μg/mL)											
		**ALA**		**EPA**		**DHA**		**LA**		**AA**		**ω-6/ω-3 ratio[a]**	
		**ρ**	***p***	**ρ**	***p***	**ρ**	***p***	**ρ**	***p***	**ρ**	***p***	**ρ**	***p***
**UPDRS I**	**Intellectual impairment**	0.101	0.552	0.221	0.189	**0.441**	**0.006**	-0.147	0.386	**0.481**	**0.003**	**-0.351**	**0.033**
	Thought disorder	-0.044	0.794	0.209	0.215	0.263	0.116	-0.194	0.251	0.089	0.601	-0.244	0.145
	**Depression**	**-0.349**	**0.034**	-0.176	0.299	-0.066	0.699	**-0.424**	**0.009**	-0.178	0.293	-0.048	0.777
	**Motivation/initiative**	-0.178	0.291	-0.086	0.611	0.019	0.910	-0.114	0.502	**0.376**	**0.022**	-0.071	0.675
**UPDRS II**	Speech	0.220	0.192	-0.083	0.627	-0.128	0.449	0.127	0.455	0.019	0.912	-0.073	0.670
	**Salivation**	-0.185	0.272	0.025	0.883	**0.325**	**0.049**	-0.185	0.274	**0.515**	**0.001**	-0.113	0.507
	**Swallowing**	-0.192	0.254	-0.122	0.473	0.314	0.058	**-0.358**	**0.030**	**0.384**	**0.019**	-0.183	0.279
	**Handwriting**	0.031	0.854	0.240	0.152	0.266	0.112	-0.007	0.967	**0.361**	**0.028**	-0.247	0.141
	Cutting food and handling utensils	-0.023	0.891	0.141	0.407	0.105	0.538	-0.138	0.414	-0.039	0.818	-0.057	0.739
	Dressing	0.070	0.679	-0.055	0.745	-0.285	0.087	0.086	0.614	-0.119	0.481	0.159	0.346
	Hygiene	-0.066	0.699	0.184	0.275	0.078	0.645	-0.101	0.551	0.162	0.338	-0.119	0.484
	Turning in bed and adjusting bed cloths	-0.225	0.180	-0.250	0.136	-0.153	0.365	-0.059	0.728	0.101	0.552	0.132	0.436

	Falling (unrelated to freezing)	0.057	0.739	0.234	0.163	0.106	0.531	-0.090	0.597	-0.065	0.704	-0.239	0.155
	Freezing when walking	-0.044	0.797	0.112	0.509	0.103	0.542	-0.032	0.850	-0.078	0.646	-0.133	0.432
	Walking	-0.153	0.366	-0.137	0.419	-0.066	0.698	-0.301	0.071	-0.042	0.803	0.004	0.983
	Tremor	-0.136	0.423	0.174	0.302	0.017	0.918	-0.208	0.217	-0.156	0.356	-0.110	0.515
	Sensory complaints related to parkinsonism	0.008	0.961	0.158	0.350	0.153	0.365	0.039	0.818	0.000	0.999	-0.090	0.597
**UPDRS III**	**Speech**	-0.116	0.493	0.144	0.397	0.080	0.637	**-0.348**	**0.035**	-0.028	0.870	-0.206	0.221
	Facial expression	0.074	0.662	-0.158	0.349	-0.027	0.873	-0.104	0.541	-0.164	0.331	-0.007	0.967
	Tremor at rest	0.034	0.842	-0.065	0.702	0.061	0.721	-0.123	0.470	0.144	0.394	-0.176	0.298
	Action or postural tremor of hands	-0.123	0.469	0.037	0.827	-0.168	0.322	-0.278	0.096	-0.197	0.243	-0.054	0.749
	**Rigidity**	-0.278	0.096	-0.218	0.194	0.002	0.993	**-0.352**	**0.033**	-0.074	0.662	-0.042	0.804
	**Finger taps**	-0.141	0.404	-0.272	0.104	-0.248	0.140	-0.143	0.398	**-0.358**	**0.030**	0.049	0.772
	Hand movements	-0.113	0.507	-0.085	0.617	0.112	0.510	-0.053	0.753	0.117	0.492	-0.049	0.772
	Rapid alternating movements of hands	-0.050	0.770	0.030	0.862	0.099	0.562	-0.127	0.454	-0.195	0.246	-0.145	0.391
	Leg agility	-0.161	0.342	0.045	0.790	0.177	0.295	-0.168	0.321	0.207	0.218	-0.141	0.405
	**Arising from chair**	**-0.389**	**0.017**	-0.225	0.180	-0.006	0.972	**-0.401**	**0.014**	-0.006	0.974	0.110	0.515
	Posture	-0.069	0.685	0.097	0.567	0.081	0.635	-0.156	0.357	0.199	0.238	-0.159	0.349
	**Gait**	-0.252	0.132	-0.196	0.244	-0.066	0.697	**-0.427**	**0.008**	-0.097	0.567	0.063	0.709

	Postural stability	-0.151	0.373	0.038	0.825	-0.031	0.857	-0.275	0.100	-0.117	0.491	-0.055	0.747
	Body bradykinesia and hypokinesia	-0.151	0.372	0.009	0.957	-0.079	0.643	-0.198	0.240	-0.282	0.091	-0.052	0.761

The *p*-values marked in bold indicate statistical significance.

ρ: Spearman's rank correlation coefficient after adjusted for age or sex.

[a]ω-6/ω-3 ratio = (LA+AA)/(ALA+EPA+DHA)

Abbreviations: UPDRS, Unified Parkinson's Disease Rating Scale; PUFAs, polyunsaturated fatty acids; ALA, alpha-linolenic acid; EPA, eicosapentaenoic acid; DHA, docosahexaenoic acid; LA, linoleic acid; AA, arachidonic acid
